# God does not play dice, and neither does CRISPR/Cas9

**DOI:** 10.1093/nsr/nwy156

**Published:** 2019-01-22

**Authors:** Chengzu Long

**Affiliations:** Division of Cardiology, New York University School of Medicine, USA

The current dogma is that Cas9-generated double-strand breaks repaired in a template-free manner lead to imprecise insertion/deletion (indel) mutations, and that only template-mediated homology-directed repair (HDR) can facilitate precise modification. Hence, the repair products of non-homologous end joining (NHEJ) are considered heterogeneous in their outcome, and generally dismissed as random by nature. With this understanding of NHEJ, it has long been used only for imprecise gene disruption, rather than deployed as a precise and predictable means for genome editing. It is also conventionally accepted that SpCas9—a variant that has been studied in extensive detail—cleaves the genomic target strand and generates blunted ends. Wu and his team [1] break with these two previously accepted dogmas, first by demonstrating that SpCas9-mediated template-free nucleotide insertions are precise and predictable, and second by convincingly proving that SpCas9 can also generate staggered ends with 1- to 3-nt overhangs at the 5’ end using a paired guide ribonucleic acid (RNAs) approach. These findings were independently confirmed by other groups using high-throughput machine learning models [2,3] and establish a novel perspective that informs the design of a wider breadth of genome-editing strategies predicated on a predictable and template-free correction of pathogenic mutations.

**Figure 1.**
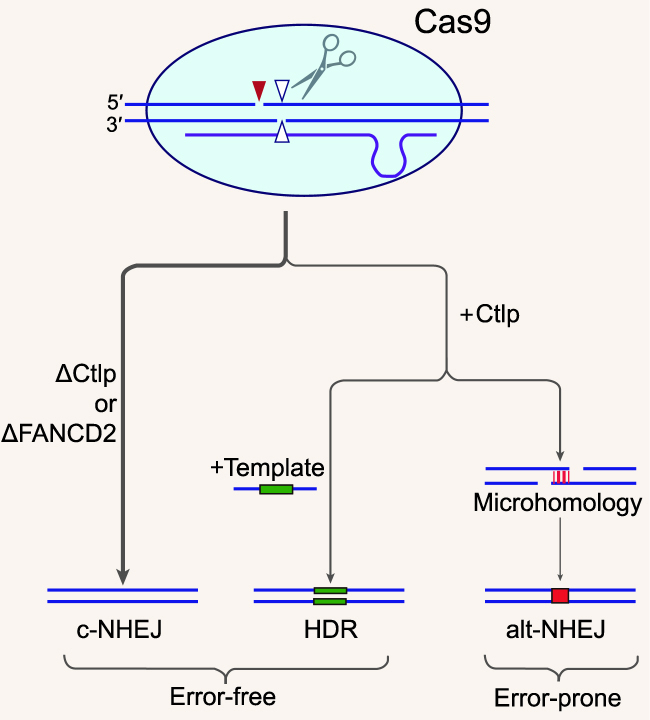
Illustration of the roles of FANCD2 or CtIP in Cas9-mediated editing.

Since the activities and outcomes of Cas9-mediated editing were previously thought to be unpredictable, most researchers would typically design and test multiple guide RNAs targeting the same locus. Only if they got ‘lucky’ would this approach yield a desirable editing outcome. Much like rolling a dice, you will most certainly end up with a ‘number’, though not necessarily the one that wins the game. To en-hance precise DNA-fragment deletion repaired by the error-free canonical NHEJ (c-NHEJ) pathway, Shou *et al.* systematically screened the proteins in the alternative NHEJ (alt-NHEJ) pathway and found that disrupting CtIP or FANCD2—two proteins facilitating the resection and/or annealing of DNA double-strand breaks in alt-NHEJ and HDR—significantly increases the propensity for precise ligation through c-NHEJ. Conversely, other groups have also found that competitive DNA-repair pathways, alt-NHEJ and HDR, could be stimulated by overexpression of CtIP [4,5] (see Fig. 1).

In this flurry of mechanistic studies, investigators comprehensively examined indel patterns at junctions corresponding to the targeted CRISPR cut site and found that nucleotide insertion sites are nonrandom and in fact depend on DNA cut sequences of each guide RNA. This strongly suggests that Cas9-mediated nucleotide insertions can be predictable, and result from previously unappreciated observation of non-blunted ends generated by SpCas9. Using this new finding, researchers may be able to design better guide RNAs that have a higher chance of achieving their desired editing outcomes.

Looking at the bigger picture, the rapidly advancing field of CRISPR/Cas9-mediated genome editing is most certainly revolutionizing approaches in elucidating gene functions and correcting disease-causing mutations. Despite the huge successes of genome modification *in vitro*, reaching a level of efficacy and safety appropriate for disease therapies so that such approaches can be fully translatable to patient applications relies heavily on a better prediction of editing events in the human body—a game of dice that is still largely unfathomed. These most recent findings represent a consequential step forward in the right direction—in increasing the predictability and thereby safety of CRISPR-mediated genome-editing technologies. Hopefully, in the near future, when we are rolling the dice, we can be near certain of the results and win the game in fighting against devastating human diseases.
